# An AI-Application-Oriented In-Class Teaching Evaluation Model by Using Statistical Modeling and Ensemble Learning †

**DOI:** 10.3390/s21010241

**Published:** 2021-01-01

**Authors:** Junqi Guo, Ludi Bai, Zehui Yu, Ziyun Zhao, Boxin Wan

**Affiliations:** 1 School of Artificial Intelligence, Beijing Normal University, Beijing 100875, China; guojunqi@bnu.edu.cn (J.G.); YUZE0004@e.ntu.edu.sg (Z.Y.); 202021210011@mail.bnu.edu.cn (Z.Z.); 202021210002@mail.bnu.edu.cn (B.W.); 2 Engineering Research Center of Intelligent Technology and Educational Application, Ministry of Education, Beijing 100875, China

**Keywords:** in-class teaching evaluation, index system, statistical modeling, ensemble learning, artificial intelligence (AI)

## Abstract

In-class teaching evaluation, which is utilized to assess the process and effect of both teachers’ teaching and students’ learning in a classroom environment, plays an increasingly crucial role in supervising and promoting education quality. With the rapid development of artificial intelligence (AI) technology, the concept of smart education has been constantly improved and gradually penetrated into all aspects of education application. Considering the dominant position of classroom teaching in elementary and undergraduate education, the introduction of AI technology into in-class teaching evaluation has become a research hotspot. In this paper, we propose a statistical modeling and ensemble learning-based comprehensive model, which is oriented towards in-class teaching evaluation by using AI technologies such as computer vision (CV) and intelligent speech recognition (ISR). Firstly, we present an index system including a set of teaching evaluation indicators combining traditional assessment scales with new values derived from CV and ISR-based AI analysis. Next, we design a comprehensive in-class teaching evaluation model by using both the analytic hierarchy process-entropy weight (AHP-EW) and AdaBoost-based ensemble learning (AdaBoost-EL) methods. Experiments not only demonstrate that the two modules in the model are respectively applicable to the calculation of indicators with different characteristics, but also verify the performance of the proposed model for AI-based in-class teaching evaluation. In this comprehensive in-class evaluation model, for students’ concentration and participation, ensemble learning module is chosen with less root mean square error (*RMSE*) of 8.318 and 9.375. In addition, teachers’ media usage and teachers’ type evaluated by statistical modeling module approach higher accuracy with 0.905 and 0.815. Instead, the ensemble learning approaches the accuracy of 0.73 in evaluating teachers’ style, which performs better than the statistical modeling module with the accuracy of 0.69.

## 1. Introduction

With the development of modern high-technology, the field of education has ushered in a major change in the combination of traditional form and information technology, which is considered as educational informatization.

From a general survey of relevant documents (e.g., National Educational Technology Plan 1996–2016) [1], it is easy to find that the educational informatization in the United States has gone through several stages including infrastructure construction, digital resources improvement, and the training of teachers’ application ability. At present, it has entered the intelligent application stage of educational informatization. Some other countries also have many educational informatization application cases [2,3]. For instance, robots are utilized in Japanese high school to assist campus management and programming education [4]. A large quantity of E-learning resources is added to STEAM(Science, Technology, Engineer, Arts and Math) education in Korea [5]. Australia combines virtual reality technology with children’s education to pilot virtual classroom education [6]. In China, the development of education informatization has been strongly supported by national policies. According to “the 13th Five-Year-Plan of the National Education Development [7]” in 2017 and “the Education Informatization 2.0 Action Plan [8]” in 2018, it is clearly proposed that one of the major projects of educational modernization is to accelerate the process of educational informatization, aiming at smart education as its future application goal. “China Education Modernization 2035 [9]” released in 2019 also clearly pointed out that building an ensemble platform of intelligent teaching evaluation, management, and service will accelerate the development of smart education, in which in-class teaching evaluation and campus management are two representative applications. 

Considering that a classroom is the most important place for traditional teaching and learning activities, in-class teaching quality evaluation is the key aspect of the whole education quality assessment system. In a classroom, teachers are committed to planning teaching activities, while students carry out learning activities under the guidance of teachers. Whichever aspect goes wrong may affect the entire in-class performance. The method of in-class teaching evaluation has mainly experienced three stages: (1) In the early stage, investigators were organized to go into classrooms for class observation, and then evaluated in-class performance by using traditional assessment scales and questionnaires; (2)The emergence of video recording technology made it possible for some courses to use videos instead of classroom observation, yet these videos still need to be watched manually for analysis; (3) Driven by the demand for convenience, efficiency, and accuracy, emerging artificial intelligence (AI) technology makes the automatic analysis of in-class audio and video data become a reality, thus replacing the manual evaluation workloads.

For the above two traditional evaluation methods, it is indispensable to utilize some special in-class assessment scales, which mainly include five indicators: teaching objective, content, method, means, and effect. Additionally, there are multiple secondary indicators belonging to these main indicators, which make evaluation dimension cover more specific observation points and thus improve both reliability and validity of scales [10]. Besides, some evaluation scales include indicators of students’ activities in class, such as participation status, activity status, thinking status, and learning effect. All the above indicators combine teaching and learning monitoring to establish a complete index system for evaluation. For example, Chen Youqing [11] proposed a teaching evaluation index system by combining three conventional scales, which includes four first-level indicators such as the pertinence, initiative, diversity, and selectivity of learning, as well as several second-level and third-level indicators. This study quantified the assessment of in-class teaching from the perspective of “teaching evaluation by learning”. The University of California Berkeley organized students from various departments to evaluate in-class teaching quality according to curriculum design and teaching content by subjective scale scoring [12]. Stanford University sent anonymous questionnaires to students for in-class feedback and conducted one-on-one interviews after class, the results of which were evaluated by a professional evaluation agency. These traditional evaluation methods are to use manual analysis, which is inevitably influenced by subjective factors of observers. Sometimes there is a lack of enough objective data to get a reliable conclusion [13]. Furthermore, evaluators must spend a lot of manpower, material resources, and time to make in-class observation, scale scoring, and interviews, while in-class observation may even affect the normal teaching activities. Therefore, improving the traditional in-class teaching evaluation pattern has become an urgent problem in the field of education.

For AI-based evaluation methods, the use of intelligent technology to analyze in-class teaching behaviors may bring more objective, timely, and efficient results. In China, the popularization of the in-class intelligent attendance system has realized convenient attendance check-in [14]. Dai et al. [15] put forward a kind of student motion capture system based on camera, and then the report of each student’s learning interest in class can be obtained, which helped to teach students in accordance with their aptitude and to improve the teaching effect. Yi He et al. [16] proposed a lightweight convolution-neural-network (CNN) model for practical teaching evaluation, the experimental results of which confirmed the possibility of the deep learning-based models in improving evaluation performance. Samaher [17] designed a smart education environment through the combination of intelligent data analysis (IDA) and Internet of Things (IoTs) to automatically detect student violations and teacher speeches, and then automatically provided corresponding reminders to reduce manpower demand for campus management.

In this paper, we propose a statistical modeling and ensemble learning-based comprehensive model, which is oriented towards in-class teaching evaluation by using AI technologies such as computer vision (CV) and intelligent speech recognition (ISR). 

Firstly, we present an index system including a set of teaching evaluation indicators combining traditional assessment scales with new values derived from CV and ISR-based AI analysis. Generally, AI is good at visual and speech analysis, while some indicators in the traditional teaching evaluation scales cannot be calculated from AI-based video and audio analysis. On the other hand, the real in-class video and audio data may contain certain process characteristics that are not available in traditional scales. Therefore, it is necessary to design a more systematic and comprehensive index system for in-class teaching evaluation. We refine and summarize a set of teaching evaluation indicators combining traditional assessment scales with new values derived from AI analysis, which not only has the advantages of objectivity from the real data, but also retains the experience of subjective evaluation in the classical scales.

Next, we design a comprehensive in-class teaching evaluation model consisting of both the analytic hierarchy process-entropy weight (AHP-EW) and AdaBoost-based ensemble learning (AdaBoost-EL) modules. Based on human motion, emotion, speech and class assignment text obtained from the real video and audio in class by AI technology, the statistical modeling-based AHP-EW method is used to model the data at the subjective and objective levels, respectively. At the same time, the ensemble learning model based on AdaBoost is used to deeply mine the data, to establish the mapping relationship between the observed data and in-class teaching indicators.

Finally, experiments are carried on to verify the feasibility and efficiency of the proposed model. Experimental results demonstrate that the two modules in the model are respectively applicable to the calculation of indicators with different characteristics. According to performance analysis, the proposed comprehensive model combined with the refined index system has been proven to be effective for AI-based in-class teaching evaluation.

## 2. In-Class Evaluation Framework

Flanders interaction analysis system (FIAS) [18] is an in-class behavior analysis technology proposed by American in-class researcher N.A. Flanders in the 1960s, which is more meticulous and mature in the earlier speech act theory of in-class teacher-student interaction. FIAS is composed of a coding system for describing the interaction in the class, a set of prescribed standards for observing and recording codes, and a matrix table for displaying data, conducting analysis, and achieving research purposes. It is the beginning of in-class evaluation in the modern form.

With the continuous improvement of educational scientific research methods, the introduction of scientific quantitative research tools, such as coding tables, and the improvement of recording and video equipment, in-class observation methods and techniques were enriched, making it more operable. However, while quantifying in-class observations deepened the description and understanding of in-class teaching, it could not conceal its pure technical deficiencies. From the 1970s, the qualitative research method began to enter the in-class observation [19]. The complete text description presented the whole picture of the class, so that the in-class events and in-class movement that were originally stripped out could return to the situation itself. Then, researchers could use their personal experience to better understand the class. Today, two different qualitative research orientations have enriched in-class observations from different levels and directions. The combination and complementarity of the two orientations have become the mainstream trend of in-class observation development [20].

In our in-class teaching evaluation system, firstly, according to the observation record coding rules in FIAS, we sample and code the interactive behaviors in the class every three seconds, and get eight kinds of data sets as the input basis (including teachers’ movement, teachers’ emotion, teachers’ speech text, teachers’ volume and speed, teachers’ label, students’ movement, students’ emotion, students’ label). Then, according to the traditional classroom evaluation scales, a set of classroom evaluation index system suitable for AI is designed.

**Figure 1:**
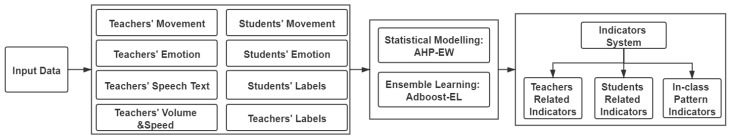
The framework of our in-class evaluation model.

In order to complete the mapping relationship between the input data and the index system, the system also includes two evaluation modules: a statistical modeling module based on AHP and entropy weight method and an ensemble learning module based on AdaBoost. By inputting the relevant characteristics of teachers’ and students’ movements and emotions, the evaluation results are calculated to complete the corresponding indicator mapping relationship. Finally, based on the results of the performance test part, the model that suits different indicators is selected to build a comprehensive in-class teaching evaluation model system. The overall structure of the system is shown in Figure 1.

## 3. Index System Design

The traditional in-class evaluation method will gain a lot of rich experience and valuable suggestions from the experts, but it will cost more time and human resources. Moreover, manual filling in the scale for in-class evaluation will inevitably introduce subjective factors, which will affect the reliability of the evaluation results. The method of AI in-class evaluation is to analyze multidimensional data such as the movement and speech of teachers and students in the class through AI technology, which can overcome the subjectivity and empirical dependence of traditional evaluation to a certain extent. Therefore, we have studied some classic in-class evaluation measurement tables, refined and summarized a set of in-class evaluation index system suitable for AI analysis.

At present, traditional in-class teaching evaluation was to fill in the evaluation scales after the expert groups listen to the class, and the main indicators were mainly used to evaluate: teaching contents, teaching goals, teaching methods, teaching attitude, teaching effect, and so on. As it was difficult for the FIAS system to carry out a more classified and detailed evaluation of activities in class, Professor Cui [21] expanded the main means of in-class observation in the in-class evaluation practices in 2012. He proposed a new way of professing in-class observation, “the Paradigm of LICC”, which was composed of four dimensions: learning, instruction, curriculum, and culture. LICC is widely used in the design of in-class teaching evaluation indicators.

**Figure 2:**
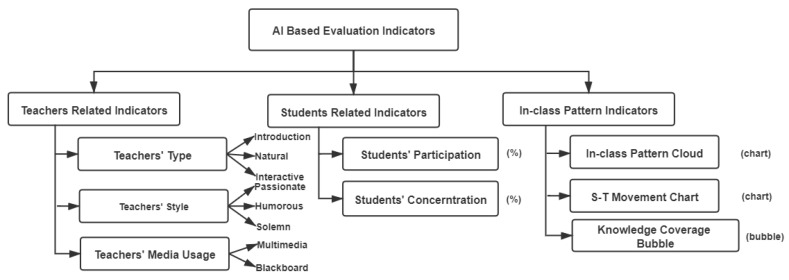
An AI (artificial intelligence)-based evaluation index system.

According to the universal indicators of LICC [22] and the existing traditional scales, we decided to divide the index system, as shown in Figure 2, into three dimensions: teacher-related indicators, student-related indicators, and in-class pattern indicators. By watching in-class videos and other observation means, teaching and research staff evaluate the in-class performance of students and teachers according to the traditional scale indicators, which is taken as label indicators. The evaluation criteria of the labels include teacher’s attainment, teacher’s method, teacher’s language ability, student’s participation, student’s concentration, in-class teaching purpose, in-class teaching content, in-class teaching effect, etc.

Except for the label indicators obtained by subjective evaluation, other indicators of teachers and students will be obtained by direct analysis of audio and video data through AI. The indicators related to the in-class pattern, such as knowledge point refine diagram and teacher–student movement analysis chart, are the overall performance results obtained through voice recognition, video analysis, and other technologies.

It is worth noting that these indicators can also be classified as score indicators and category indicators. Score indicators, including students’ concentration, students’ participation, and knowledge mastery are described by score data. And category indicators are described by categories, including teachers’ types, teachers’ style, and teachers’ media usage.

## 4. The Module of Statistical Modeling

Statistical modeling is a method that uses statistical methods to construct and analyze data and deduce the relationship between data variables [23]. With the development of technology, we can directly use computers to analyze data in batches, build statistical module, deduce general rules, and make further predictions.

### 4.1. Analytic Hierarchy Process and Entropy Weight Method

Analytic hierarchy process (AHP) [24] is to decompose each indicator of the target object into multiple constituent factors and layer them according to the relationship of the constituent factors, after which factors are analyzed layer by layer and finally the influence weights of various indicators for the target object are obtained.

**Figure 3:**
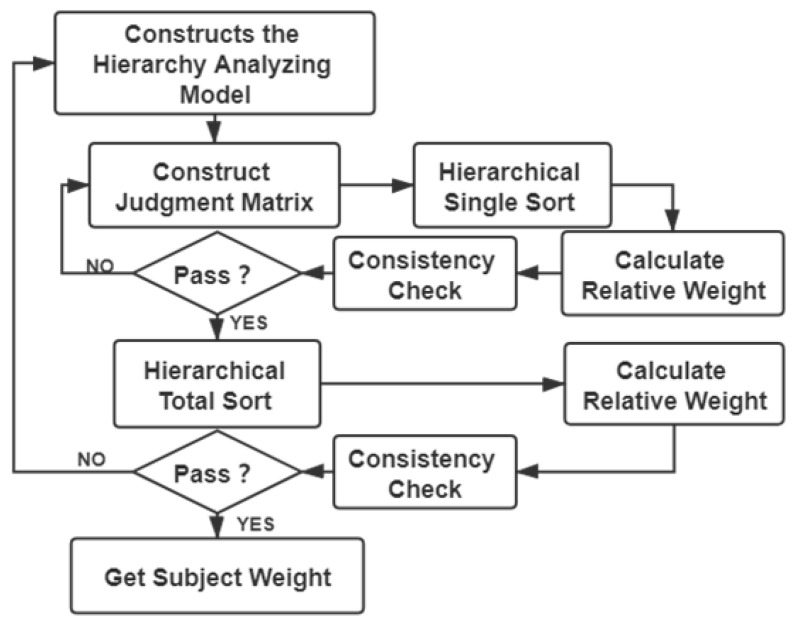
The steps to get subjective weights.

In our statistical modeling module, the steps to get subjective weights of different features for in-class teaching evaluation indicators are shown in Figure 3, which mainly includes four steps:

- Build an analytic hierarchy model module,
- Construct a judgment matrix,
- Hierarchical ordering and consistency check,
- Consistency test and get subjective weight.

Information entropy value is used to measure the magnitude of uncertainty. According to the characteristics of entropy, the degree of dispersion of a factor can be measured by the entropy value. The greater the degree of dispersion, the smaller the impact on the results [25].

Entropy weight method measures the influence of the factor on the weight of the result by calculating the entropy value of the factor [26]. Since entropy weight method uses discrete data for analysis and calculation, this method is an objective weighting method. The calculated entropy weight is the objective weight in our module [27]. The steps of calculating objective weights by entropy weight method is given in Appendix A.1 (Algorithm A1).

### 4.2. Analytic Hierarchy Process-Entropy Weight (AHP-EW) Statistical Modeling for In-Class Teaching Evaluation

**Figure 4:**
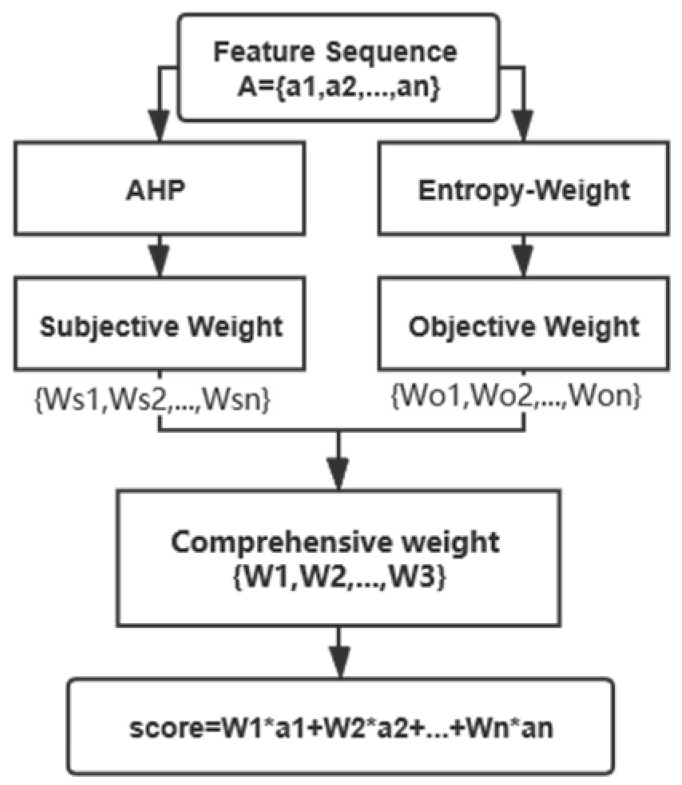
The structure of statistical modeling.

We use the analytic hierarchy process (AHP) to calculate the subjective weight of the indicators, and use the entropy weight method to calculate the objective weight of them. We calculate the decision weight from the subjective and objective aspects, and obtain the comprehensive weight based on the sequence information and strength information of the two algorithms, and then get the score or type of each indicator, which is analytic hierarchy process-entropy weight (AHP-EW) statistical modeling for in-class teaching evaluation. The structure of the statistical modeling module is shown in Figure 4.

The specific calculation steps of using the AHP-EW method for indicator mapping are as follows:

- Step 1: Determine the type of indicators and find the corresponding feature sequence.

**Figure 5:**
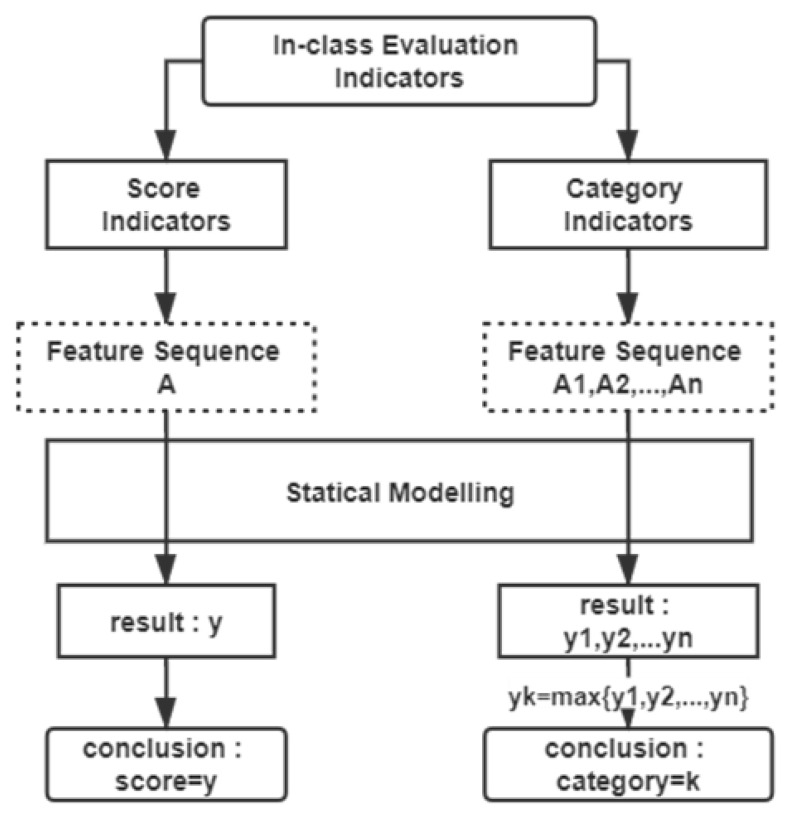
In-class teaching statistical modeling.

For evaluation of score indicators, the indicators are analysis targets of the statistical modeling and the calculated final score is the result of the evaluation. Differently, for evaluation of category indicators, the modeling targets each category of certain indicator and calculates each category’s final score. The corresponding category of the largest final score is taken as the evaluation result of a certain category indicator, as shown in Figure 5.

- Step 2: Calculate the subjective and objective weights of the features for the indicators.

According to AHP-EW method in Section 4.1, the subjective and objective weights, as well as their corresponding rankings of the features are calculated.

- Step 3: Calculate comprehensive weights by the combination weighting optimization method.

Gang Li proposed the combination weighting optimization method [28], which takes both order information and strength information of subjective and objective weights into account and achieves an optimized combination weighing performance. The formulas of the method are as follows:

(1) $$\min\sum_{i=1}^{n}{\left(\omega_{i}-\beta_{i}\right)}^{2}\;s.t.\{\begin{array}{c} \omega_{i}\geq \omega_{j}i<j\\ \alpha_{i}{}^{-}\leq \omega_{i}\leq \alpha_{i}{}^{+}\\ \sum_{i=1}^{n}\omega_{i}=1 \end{array},$$

in which n is the number of features, $\omega_{i}$ is the subjective weight of the *i*-th feature, $\beta_{i}$ is the objective weight of the *i*-th feature, and $\left[\alpha_{i}{}^{-},\alpha_{i}{}^{+}\right]$ is the reasonable value interval of the *i*-th feature.

According to the subjective and the objective weights, their reasonable value intervals of the comprehensive weights are calculated. Then, we calculate the comprehensive weights of the relevant features for the indicators by formula (1) and sorted the results.

- Step 4: Calculate each student sample’s final score of students’ concentration.

From Step 1 and Step 3, we got the normalized feature sequence and the comprehensive weight sequence of features, respectively. Further, in this step, we sum the two weighted as the final score of one indicator, which is shown as formula (2):

(2) $$score=\sum_{i=1}^{n}W_{i}\cdot a_{i},\;$$

in which *n* is the number of features, $a_{i}$ is the *i*-th normalized feature’s value, and $W_{i}$ is the comprehensive weight of the *i*-th feature.

For different indicators, the specific calculated in the statistical modeling module is described in Table 1 below. 

## 5. The Module of Ensemble Learning 

Machine learning is an approach of artificial intelligence. Through the analysis of data, the system can automatically learn the rules of data, improve from the learning experience, improve its own model learning effect, and use the rules to predict unknown data [29,30]. 

Machine learning can be divided into two categories: supervised learning and unsupervised learning. Among them, supervised learning learns a function from a given training data set. For the new data obtained, the corresponding results can be predicted according to this function. The training set of supervised learning includes input and output, that is, features and targets, and the targets in the training set are pre-annotated.

Ensemble learning is a supervised learning algorithm that has significant advantages in generalization problems and is a hot issue in the field of machine learning in recent years. The main idea is the process of combining multiple basic learners into an ensemble learner with better performance through some methods, that is to say, the generation and integration of basic learners are used to realize the construction of ensemble learners.

Ensemble learning includes many basic learners, which can be decision tree, neural network, or other machine learning algorithms. Most ensemble learning algorithms use multiple similar basic learners for homogeneous integration, while some use heterogeneous integration of multiple different basic learners [31].

At present, the commonly used ensemble learning models are divided into bagging, boosting, stacking, and blending, and the boosting algorithm is the most influential algorithm. In 1990, Schapire proved a conclusion with a constructive method [32]. “According to the classification error rate in the training process, the distribution of training subsets is constantly updated to make the learning process pay more attention to those samples that are difficult to classify. As long as we find a basic learner which is slightly better than a random guess, we can construct it into an ensemble learner with arbitrary precision.” This construction process is the core of the boosting algorithm.

### 5.1. AdaBoost

Adaptive boosting algorithm (AdaBoost) is the most classic boosting algorithm, which is an iterative algorithm proposed by Freund and Schapire in 1995 to improve the accuracy of arbitrary learners [33]. The core idea of the AdaBoost algorithm is to train different basic learners according to different subsets of the same training sample set, and then assign weights to each basic learner according to the classification error rate, and then combine them into a strong classifier.

AdaBoost can be used to solve classification problems and regression problems. It is one of the best supervised learning algorithms with the advantages of easy operation, high precision, flexible selection of basic learners, and less over fitting [34,35]. So, we choose AdaBoost as the algorithm of ensemble learning in the teaching evaluation model.

*AdaBoost includes four steps: (1) Preparing the data set, (2) Initialize training set weight, (3) Training the weak learner, (4) Combining the weak learner into a strong learner. The specific learning process is given in the Appendix A.2 (Algorithm A2)*.

### 5.2. Adaboost-Ensemble Learning (Adaboost-EL) for In-Class Teaching Evaluation

**Figure 6:**
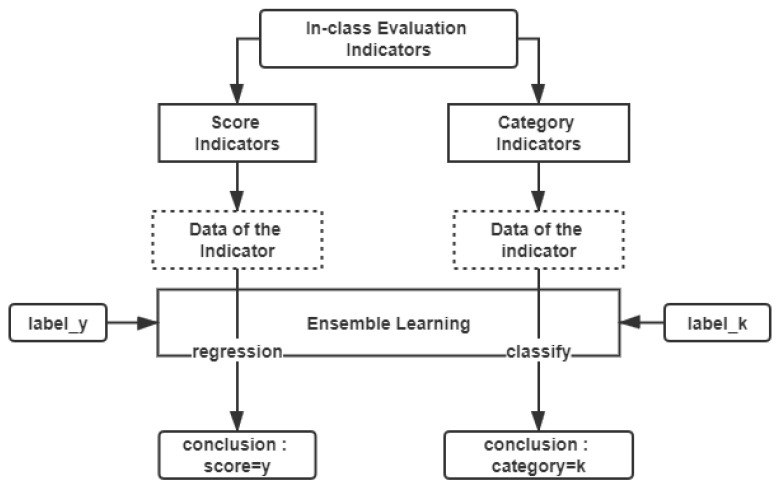
The process of ensemble learning module.

The process of the AdaBoost algorithm-based ensemble learning (AdaBoost-EL) module is presented in Figure 6.

Based on the above, we choose the AdaBoost algorithm to construct the ensemble learning module for the in-class teaching evaluation. The steps for using the AdaBoost-EL method to evaluate indicators are as follows:

- Step 1: Determine the type of the indicator and set the corresponding input data for ensemble learning module.

Before using the ensemble learning module for evaluation, it is still necessary to determine the specific types of indicators. Different types of indicators use different algorithms. For the score indicators, such as students’ concentration in class, students’ participation, etc., we build the module based on the AdaBoost regression algorithm. For the category indicators, such as teachers’ type, teachers’ style, teachers’ media usage evaluation and so on, the model is constructed based on the AdaBoost classification algorithm.

- Step 2: Construct the ensemble learning module and adjust its parameters.

According to the algorithm selected in Step 1, different loss functions and the number of basic learners is set, respectively. Experiments are carried out with the control variable method. Then, we calculate the corresponding error rate. The parameters and the loss function are determined according to the minimum error rate criterion. 

The root mean square error (*RMSE*) is usually used to measure the difference between the predicted value and the real value in the model, that is, the error rate. The smaller the value is, the closer the predicted value is to the real value, and the better the effect will be.

For different indicators, the specific calculation instructions using the ensemble learning module are shown in Table 2.

## 6. Experiment and Performance Analysis

### 6.1. Input Data

Our data set is extracted and sorted from the outcome data set of four relative research projects of our partner—Hangzhou Hikvision Digital Technology Co., Ltd., China, which are (1) ‘Machine Vision-Based Video Recognition for In-Class Movement’, (2) ‘Super Large-Scale Vocabulary Speech Recognition in Classroom Scenarios’, (3) ‘Voiceprint Recognition in Classroom Scenarios’, and (4) ‘Far Field Pickup in Classroom Scenarios’. Original data come from a real smart classroom environment deployed by Hangzhou Hikvision Digital Technology Co. In this smart classroom, there is a pickup for voice acquisition, and two cameras recording videos for teachers and students, respectively.

List of data collection equipment in the real classroom is given in Appendix B.

During the experiment, a total of 300 student samples and 200 teacher samples were selected, and the audio and video data in the class were sampled every three seconds to obtain five types of data, such as students’ movement, students’ emotion, teachers’ movement, teachers’ emotions, teacher volume, and speech speed and through the speech recognition technology to obtain the teacher speech text data of the whole class. The student label data are obtained according to the after-class test results, and the teacher label data are obtained through the evaluation of the researchers.

The input data can be divided into sequential data (students’ movement, students’ emotion, teachers’ movement, teachers’ emotion, teachers’ volume, teachers’ speed) and non-sequential data (teachers’ speech text). For sequence data, the frequency and average duration of statistics need to be counted. Additionally, for non-sequence data, word frequency statistics need to be done.

The collection methods and content of the 8 data categories are shown as Table 3:

### 6.2. Performance Analysis Indicators

In order to objectively evaluate the “statistical modeling” and the “ensemble learning model” and select the best one with better performance, we designed several commonly used performance analysis indicators.

The specific meanings and mathematical representation of five performance indexes are as follows:

(1) Root mean square error (*RMSE*):

(3) $$RMSE=\sqrt{\frac{1}{N}\sum_{i=1}^{N}{\left(y_{i}-\hat{y_{i}}\right)}^{2}}.\;$$

*RMSE* is to measure the deviation of the predicted value from the true value in the regression questions. The less the *RMSE* is, the less the deviation of the predicted value is to the true value, which also means a better performance of the regression model.

(2) Accuracy (*Accu.*):

(4) $$Accu.=\frac{n_{correct}}{N}.\;$$

Accuracy is the proportion of correctly classified samples to the total number of samples (*N*).

(3) Confusion Matrix

Confusion Matrix is a matrix showing the forecast and the actual number of positive and negative classes. It is convenient to calculate *P*, *R*, *F*1 through this.

Table 4 below is an example of a confusion matrix.

So, *TP* is the number of true positive classes, that is, the prediction and the actual label are both positive; *FN* is the number of false negative classes, that is, the prediction is negative and the actual label is positive; *FP* is the number of false positive classes, that is, the prediction is positive and the actual label is negative; *TN* is the number of true negative classes, that is, the prediction and the actual label are both negative.

(4) Precision (*P*)

Precision is the proportion of true positive in all the results predicted to the positive:

(5) $$P=\frac{TP}{TP+FP}.$$

(5) Recall (*R*)

Recall is the proportion of positive classes found by the classifier in all positive classes:

(6) $$P=\frac{TP}{TP+FN}.$$

(6) F1_score (*F*1)

F1_score is the harmonic mean of *P* and *R*:

(7) $$F1=\frac{2\times P\times R}{P+R}=\frac{2\times \mathrm{TP}}{N+\mathrm{TP}-\mathrm{TN}}.$$

(7) Macro_Precision (*M_P*)

Macro_Precision is the arithmetic average of the precision of each category (when there are more than 2 categories), which is to measure the global precision of the algorithm:

(8) $$M\_P=\frac{1}{n}\sum_{i=1}^{n}P_{i}.$$

(8) Macro_Recall (*M_R*)

Macro_Recall is the arithmetic average of the recall of each category (when there are more than 2 categories), which is to measure the global recall of the algorithm:

(9) $$M\_R=\frac{1}{n}\sum_{i=1}^{n}P_{i}.$$

(9) Macro_F-measure (*M_F*1)

Macro_F-measure is the Harmonic mean of *M_P* and *M_R*:

(10) $$M\_F1=\frac{2\times M\_P\times M\_R}{M\_P+M\_R\;}.$$

### 6.3. Model Construction and Parameter Selection

#### 6.3.1. The Example of Statistical Modeling Module

Now, we take students’ concentration as an example for further analysis.

- Step 1: Determine the type of the indicator and find the corresponding feature sequence.

According to Section 3, students’ concentration is classified as a score indicator. In addition, from data preprocessing, 20 students’ features such as frequency and average duration of students’ movement and emotion are extracted from original students’ data set. Then, we select 11 features related to students’ concentration and determine their positive and negative effects to get a directional feature sequence, after which the feature sequence is normalized to the range [0,100].

- Step 2: Calculate the subjective and objective weights of 11 features for students’ concentration

According to AHP and the entropy-weight method in Section 4, the subjective and objective weights, as well as their corresponding rankings of 11 features are calculated, as shown in Table 5.

Among them, X1–X11 are the frequency of “looking up to listen to lectures”, the average duration of “looking up to listen to lectures”, the average duration of “looking down to take notes”, the frequency of “lying on the table”, the average duration of “lying on the table”, the frequency of “looking aside”, the average duration of “looking aside”.

- Step 3: Calculate comprehensive weights by the combination weighting optimization method.

Using the formula (1), we take both order information and strength information of subjective and objective weights into account and achieve an optimized combination weighing performance. 

Then, according to the subjective and objective weights of 11 features in column ②–⑤ of Table 5, their reasonable value intervals of comprehensive weights are listed in column ⑥. Then, we calculate the comprehensive weights and rankings of 11 features for students’ concentration by formula (7). The results are shown in column ⑦–⑧ of Table 5.

- Step 4: Calculate each student sample’s final score of students’ concentration.

From Step 1 and Step 3, we get the normalized feature sequence and the comprehensive weight sequence of features, respectively. Further, in this step, we sum the two weighted as the final score of each sample’s students’ concentration, which is shown as formula (2).

#### 6.3.2. The Example of Ensemble Learning Module

Like the statistical modeling module, we also introduce the construction of ensemble learning module through an example of students’ concentration.

- Step 1: Determine the type of the indicator and set the corresponding input data for the AdaBoost-EL method.

Students’ concentration is classified as a score indicator. In addition, 20 features extracted from original students’ data set in data preprocessing constitute the input data for the AdaBoost-EL method with 300 student samples and 20 features of each sample. Then, we randomly select 40% as the testing set and 60% as the training set.

- Step 2: Construct the ensemble learning module and adjust its parameters.

Because students’ concentration is a score indicator, we choose CART(Classification and Regression Trees) regression tree as the basic learner, and set different loss functions (linear loss, square loss, and exponential loss) and the number of basic learners (30,50,70,100), respectively, with the idea of using control variables to carry on the experiment. Then, we calculate the corresponding root mean square error (*RMSE*) to evaluate the results. Among them, the *RMSE* is used to measure the gap between the predicted value and the real value in the model. The smaller the value is, the closer the predicted value is to the real value, and the better the effect is. The test results are as follows.

It can be seen from the results, shown in the Table 6, that when predicting the students’ concentration, the *RMSE* is the smallest when selecting 70 basic learners and linear loss functions, which is 9.3749, and the prediction effect of ensemble learning is the best. 

**Figure 7:**
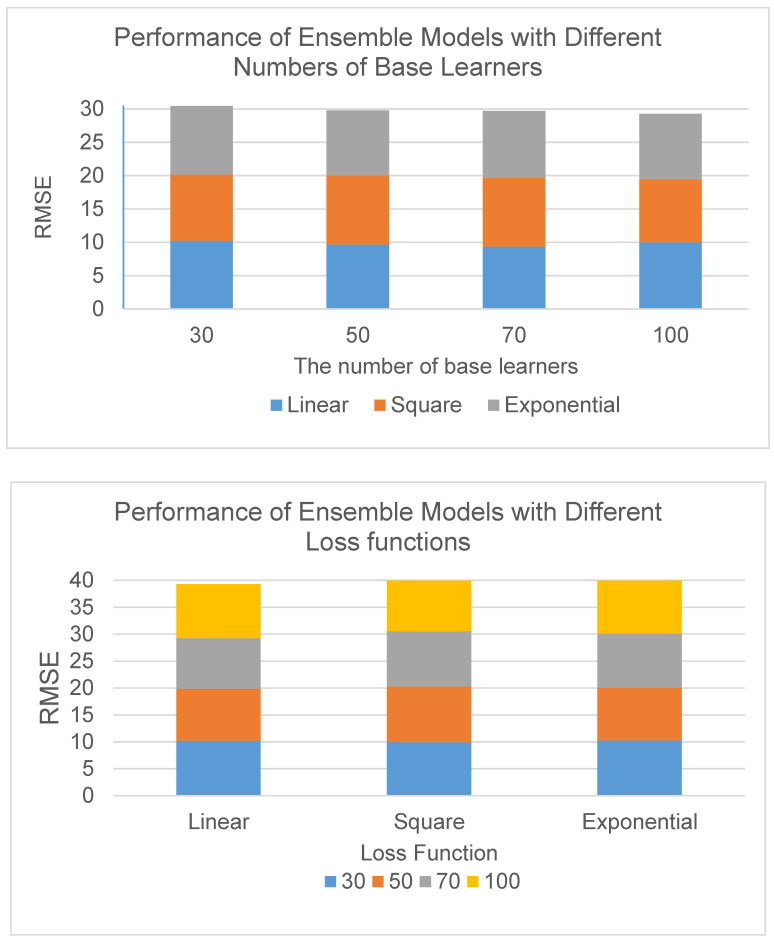
The overall result chart of ensemble learning module with different parameters.

From the overall result chart (Figure 7), it can be found that the overall test result of the linear loss function is better than the other two loss functions, and as the number of basic learners increases, the smaller the overall *RMSE*, that is, the better the prediction effect.

### 6.4. Performance Analysis

**Figure 8:**
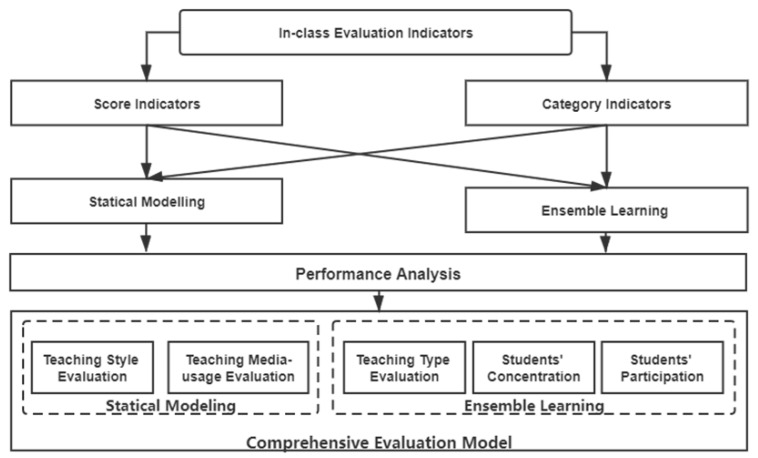
Process chart of the performance analysis experiment.

In this section, aiming to construct a better comprehensive evaluation model through comparing and combining statistical modeling and ensemble learning, we tested both modules’ performances in different in-class teaching evaluation indicators by the performance analysis indicators introduced in the above Table 6. To thoroughly compare the proposed statistical modeling and ensemble learning modules, we apply them to the evaluation of each score indicators and each category indicators separately, which is shown in Figure 8.

#### 6.4.1. Performance Analysis of Statistical Modeling Module

**Figure 9:**
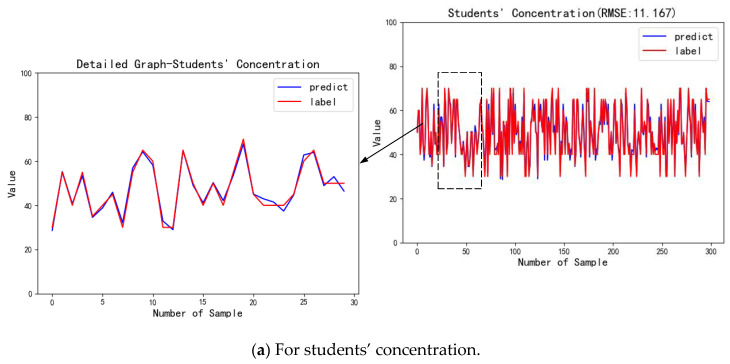

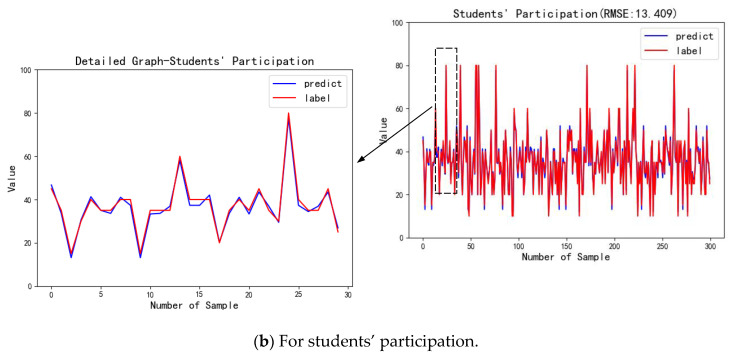
Performance analysis for score indicators evaluated by the statistical modeling module.

For the score indicators, students’ concentration and students’ participation, the evaluation result is continuous. Therefore, *RMSE* is used to perform the AHP-EW method, and the prediction results are compared with the label data, as shown in Figure 9.

According to the performance analysis, we obtained the *RMSE* of the statistical modeling module for score indicators, which is shown in Table 7.

From the above figures, the AHP-EW does well both in the two score indicators, which both have high fitting degrees between the predicted value and the label value. It can be calculated that the *RMSE* between the predicted value and the label value of students’ concentration was 11.167, and the *RMSE* of students’ participation was 13.409. Compared between the two indicators, the AHP-EW does better in students’ concentration than the other one.

**Figure 10:**
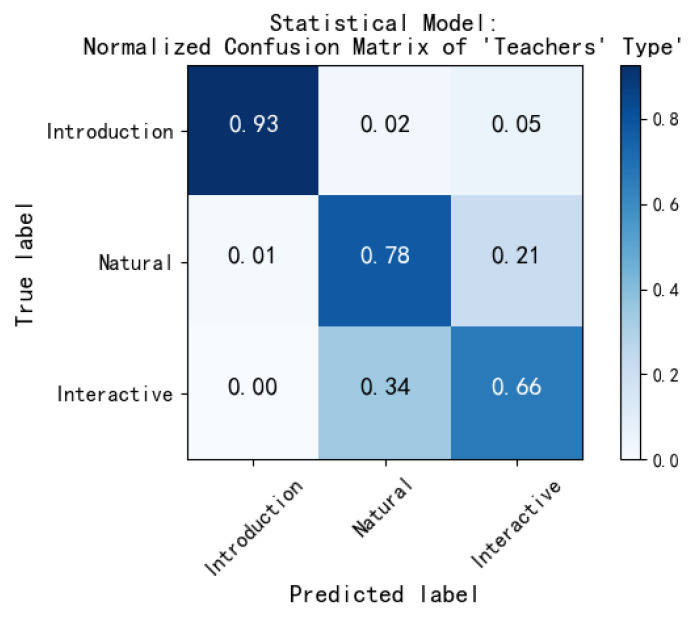

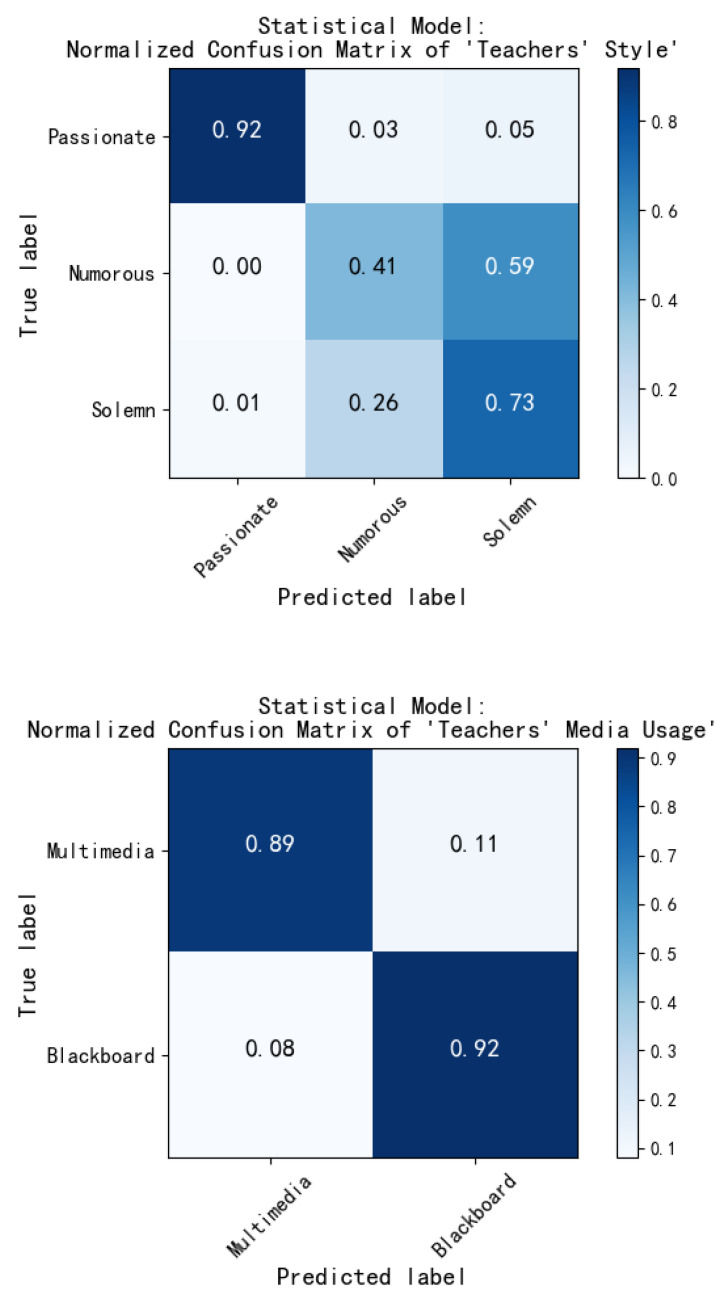
Confusion matrix for category indicators evaluated by the statistical modeling module.

For the category indicators, the evaluation of teachers’ type, teachers’ style, and teachers’ media usage, we obtain the normalized confusion matrix under the statistical modeling, as shown in Figure 10.

According to the confusion matrix, it is easy to calculate the performance parameters of each category of three indicators: The precision, recall, F1-score value of each class, and accuracy, *M_P*, *M_R*, *M_F*1 to evaluate the global performance of statistical modeling, as shown in Table 8.

According to the above table, among the various categories’ evaluation of teachers’ type, “indoctrination” performs well, and the rest is mediocre, and the overall performance of the method is in the evaluation of teachers’ style, the performance of “passionate” is better than that of “humorous” and “solemn”. The overall performance of the model for this indicator is relative. There are only two categories in the teachers’ media usage, and the overall performance is also good.

#### 6.4.2. Performance Analysis of Ensemble Learning Module

Similar to the performance analysis of the statistical modeling module, it still distinguishes score indicators and category indicators.

**Figure 11:**
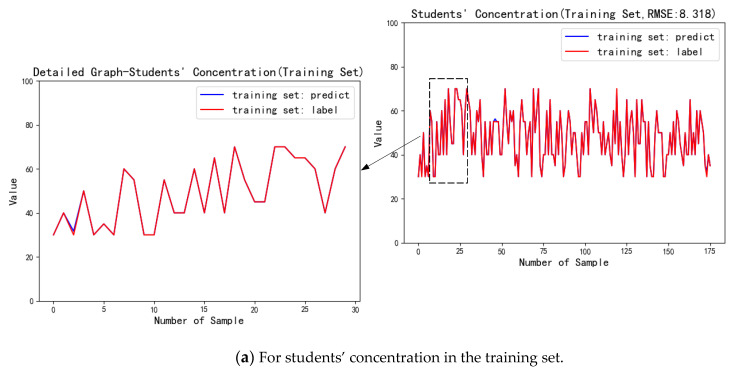

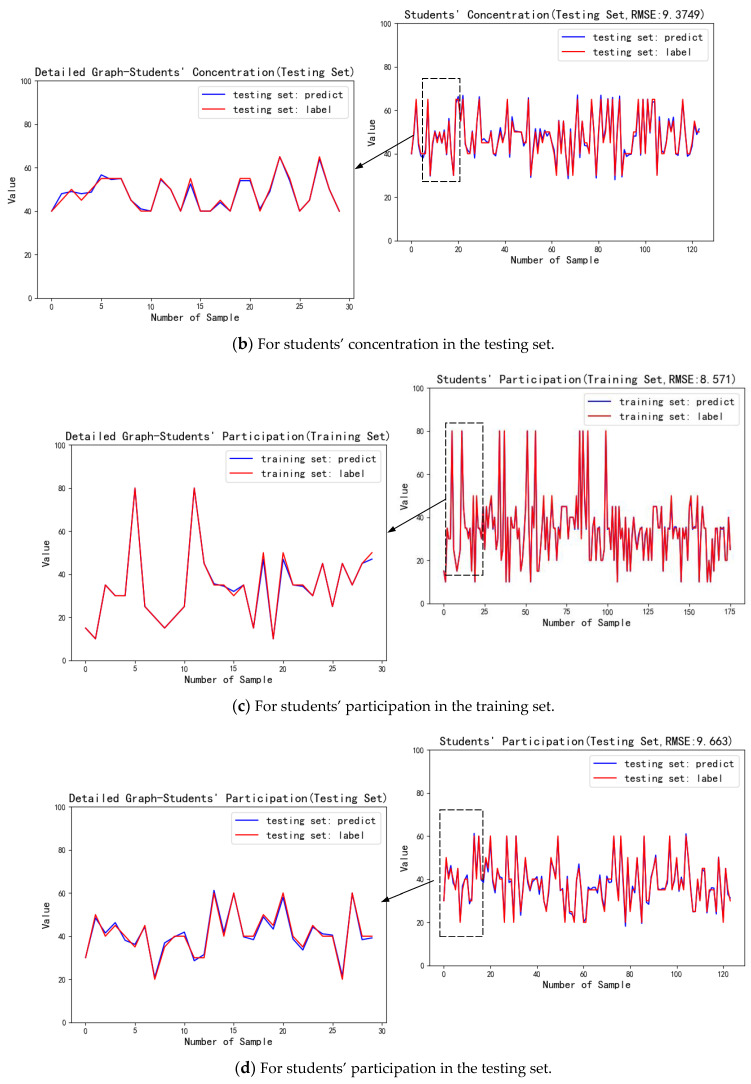
Performance analysis for score indicators evaluated by the ensemble learning model.

For the score indicators, students’ concentration and students’ participation, the evaluation result is continuous, so we use the same analysis indicator, *RMSE*, to calculate the accuracy of the AdaBoost-EL method, and draw the prediction results of the model and compare with the label data, as shown in Figure 11.

According to the performance analysis, we obtained the *RMSE* of the ensemble learning module for score indicators, which is shown in Table 9.

The charts above are the comparison of the predicted results and label data of the students’ concentration in the training set and the test set. From the fitting degree of the chart, we can find that the ensemble learning model has a good prediction effect both on the training set and test set. Among them, the *RMSE* of prediction results and label data on the training set is 8.318, and the *RMSE* on the test set is 9.3749.

The following charts are the comparison of the predicted results and label data of the students’ participation in the training set and the test set. Among them, the *RMSE* of prediction results and label data on the training set is 8.571, and on the testing set is 9.663. The fitting degree is slightly lower, but it is still higher than the statistical model.

**Figure 12:**
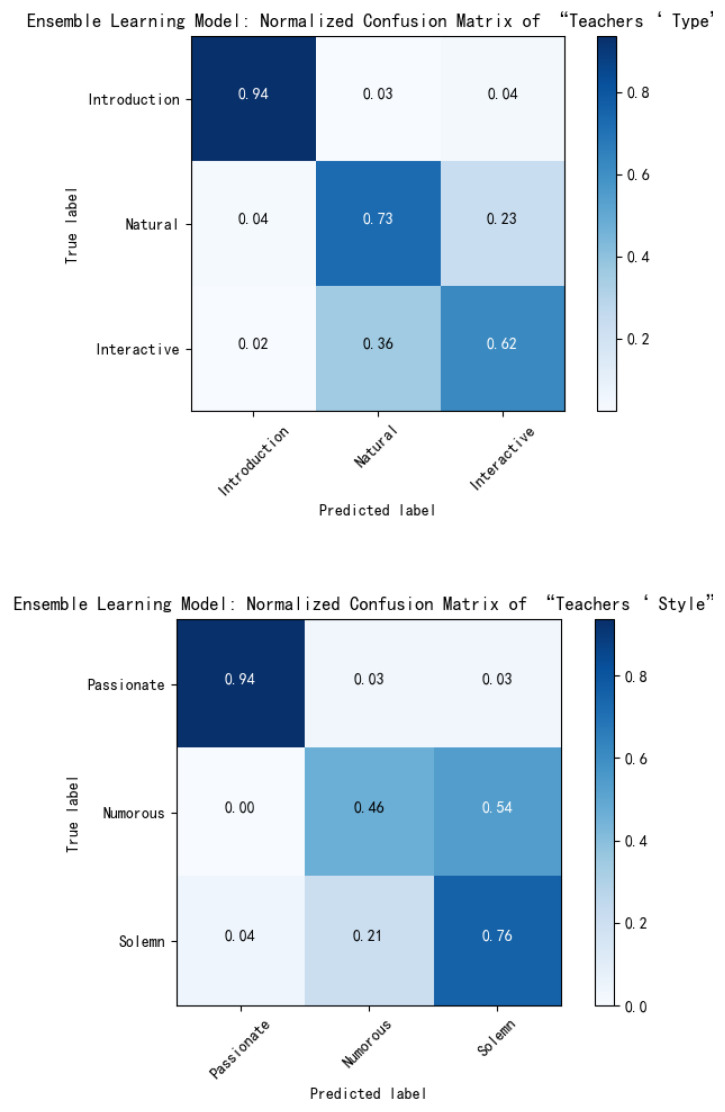

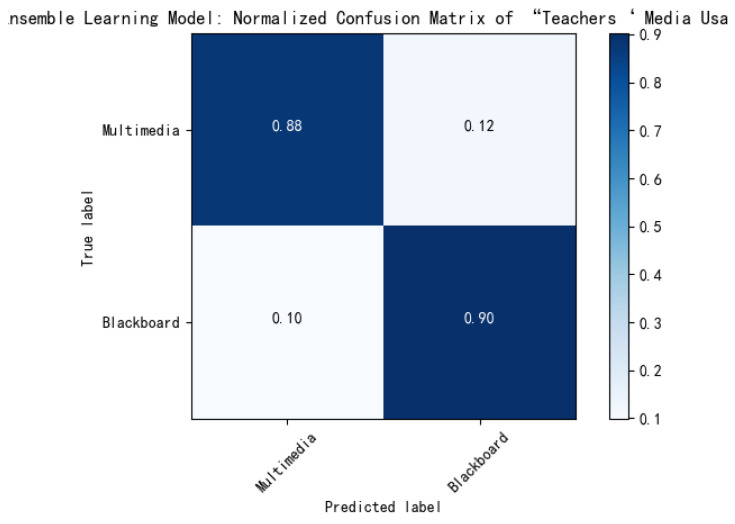
Confusion matrix for category indicators evaluated by the ensemble learning module.

Similarly, for the category indicators, teachers’ type, teachers’ style and teachers’ media-usage, the normalized confusion matrix of the three indicators on the ensemble learning model is shown in Figure 12.

According to the confusion matrix, the performance parameters of the three indicators in the ensemble learning model are calculated: *P*, *R*, *F*1 of each class, and accuracy, *M_P*, *M_R*, *M_F*1, which are used to evaluate the global performance of the ensemble learning model, as shown in Table 10.

According to the above table, it can be concluded that the performance of teachers’ type is better for “indoctrination”, while for the other two indicators, it is similar to the performance of the statistical model in each category.

In the evaluation of teachers’ style, the values of *P*, *R*, and *F*1 of “passive” are better than the values of “solemn”, while the performance of “humorous” is the worst.

The overall performance of ensemble learning is also better for the binary indicator, teachers’ media usage. Like the statistical model, the misjudgment rate of multimedia is higher than that of blackboard.

#### 6.4.3. Comparison between the Two Modules

To distinguish the performance of the two modules more conveniently, we summarize the performance parameters of the two modules into a table (Table 11).

**Figure 13:**
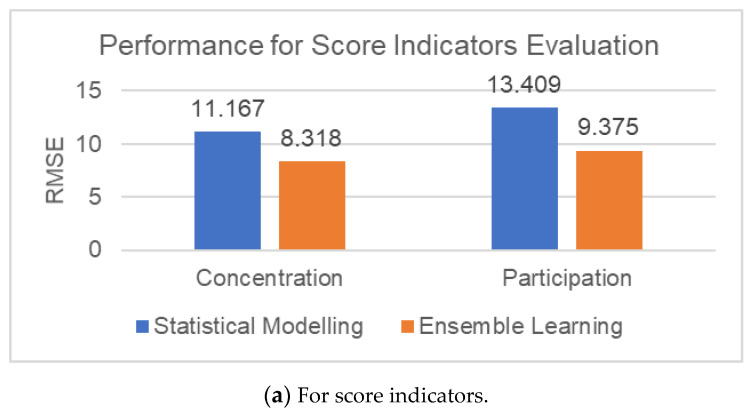

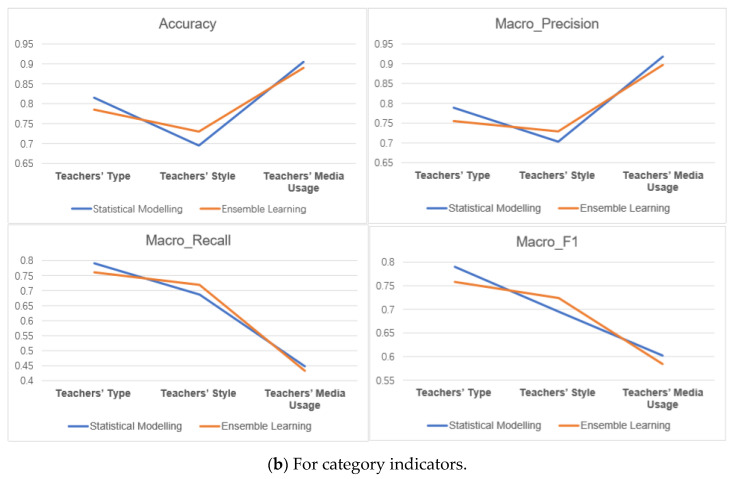
The performance for score indicators and category indicators.

The above table includes the performance of the statistical modeling and the ensemble learning modules for five indicators, teachers’ type, teachers’ style, teachers’ media usage, students’ concentration, and students’ participation. Interpreting the data into graphs can more clearly compare the performance between the two modules, as shown in Figure 13.

The first histogram is for score indicators, students’ concentration and students’ Participation, we mainly use *RMSE* to measure the error between the predicted value and the real value. In contrast, it is obvious that the *RMSE* of ensemble learning module is smaller for the two score indicators, which means the evaluation performance of the AdaBoost-EL method is more suitable.

The second group of line charts are for category indicators, teachers’ type, teachers’ style, teachers’ media usage.

According to the curve in the figure, for teachers’ style, the evaluation effect of ensemble learning module is better, which can be more targeted to the characteristics of indicators in-depth mining, using the idea of ensemble learning to enlarge the complementarity between data.

However, for the other two indicators, teachers’ type and teachers’ media usage, it is obvious that the performance of the statistical modeling is better. We can use the AHP-EW methods with a combination of subjective and objective to better extract the correlation between features.

**Figure 14:**
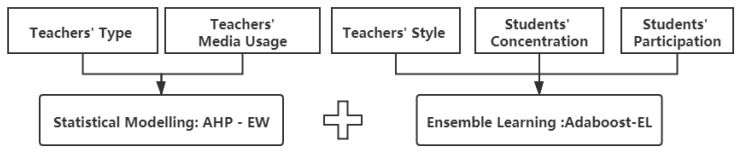
The comprehensive in-class evaluation model.

According to the performance analysis in the previous section, for different indicators, the AHP-EW method and the AdaBoost-EL method have their own advantages and disadvantages. For the proposed indicators, we choose models that perform better in the evaluation results, and combine the comprehensive in-class evaluation model, as shown in Figure 14.

## 7. Conclusions

Aiming at accelerating the development of smart education, this paper has proposed an AI-application-oriented in-class teaching evaluation model by using statistical modeling and ensemble learning.

First of all, this paper designs a more systematic and comprehensive index system for in-class teaching evaluation, including a set of teaching evaluation indicators combining traditional assessment scales with new values derived from AI analysis, which not only better suits AI analysis output, but also retains the rich experience of scales and questionnaires. Next, a comprehensive in-class evaluation model consisting of statistical modeling and ensemble learning is proposed to establish the accurate mapping relationship between the observed data and teaching evaluation indicators. The module of statistical modeling utilizes the AHP-EW method to model the subjective and objective data. Meanwhile, the module of ensemble learning employs the AdaBoost method to deeply mine the data. Experiments not only demonstrate that the two modules in the model are respectively applicable to the calculation of indicators with different characteristics, but also verify the performance of the proposed model for AI-based in-class teaching evaluation. In this comprehensive in-class evaluation model, for students’ concentration and participation, ensemble learning module is chosen with less root mean square error (*RMSE*) of 8.318 and 9.375. In addition, teachers’ media usage and teachers’ type evaluated by the statistical modeling module approach higher accuracy with 0.905 and 0. 815. Instead, the ensemble learning approaches the accuracy of 0.73 in evaluating teachers’ style, which performs better than the statistical modeling module with the accuracy of 0.69.

## Figures and Tables

**Table 1:** The calculation of indicators in the statistical modeling module.

Index	Input	Feature	Output
Students Concentration	Students’ Movement Students’ Emotion Concentration Judgment Matrix Concentration Labels	Frequency and Average Duration of 8 types of Students’ Movement; Frequency and Average Duration of 2 types of Students’ Emotion;	Score_Concentration
Students’ Participation	Students’ Movement Students’ Emotion Participation Judgment Matrix Participation Labels	Frequency and Average Duration of 8 types of Students’ Movement; Frequency and Average Duration of 2 types of Students’ Emotion;	Score_Participation
Teachers’ Type	Teachers’ Movement Teachers’ Emotion Teaching Type Labels	Frequency and Average Duration of 9 types of Teachers’ Movement; Frequency and Average Duration of 2 types of Teachers’ Emotion;	Score_ Indoctrination Score_ Natural Score_ Interactive
Teachers’ Style	Teachers’ Movement Teachers’ Emotion Teachers’ Volume and Speed Teaching Style Labels	Frequency and Average Duration of 9 types of Teachers’ Movement; Frequency and Average Duration of 2 types of Teachers’ Emotion; Mean and Variance od Teachers’ Volume and Speed	Score_Passionate Score_Humorous Score_Solemn
Teachers’ Media usage	Teachers’ Movement Media Usage Labels	Frequency and Average Duration of 9 types of Teachers’ Movement;	Score_ Multimedia Score_ Blackboard

**Table 2:** The calculation of indicators in the ensemble learning module.

Indicator	Input	Base Learners	Classification Algorithm	Output
Students’ Concentration	Students’ Movement Students’ Emotion Concentration Labels	Regression Tree		Forecast Score of Concentration
Students’ Participation	Students’ Movement Students’ Emotion Participation Labels			Forecast Score of Participation
Teachers’ Type	Teachers’ Movement Teachers’ Emotion Teaching Type Labels	Classification Tree	SAMME	Types: Indoctrination, Natural, Interactive
Teachers’ Style	Teachers’ Movement Teachers’ Emotion Teachers’ Volume and Speed Teachers’ Style Labels			Types: Passionate, Humorous, Solemn
Teachers’ Media-usage	Teachers’ Movement Media Usage Labels			Types: Multimedia, Blackboard

**Table 3:** The collection methods and content of the 8 data categories.

	Data Categories	Collection Methods	Data Content
200 teacher samples	Movement	Collect teachers’ movements per 3 s	Movement number (1–9) and corresponding time
	Emotion	Collect teachers’ emotions per 3 s	Emotion numbers (1–2) and corresponding time
	Volume and Speed	Collect teachers’ volume (dB) and speed (word per minute) per 3 s	Volume value, speed value and corresponding time
	Speech Text	The content sequence of process speech text in the whole class	Every sentence and its start and end time
	Labels	Three evaluation labels marked by experts to evaluate the teachers from the courses.	Teaching type (1–3), Teaching style (1–3), Media usage (1–2).
300 student samples	Movement	Collect students’ movements per 3 s	Movement numbers (1–8) and corresponding time
	Emotion	Collect students’ emotions per 3 s	Emotion numbers (1–2) and corresponding time
	Labels	According to the test after class and the Concentration and Participation in the whole class	Scores of the tests, Concentration and Participation in class

**Table 4:** An example of a confusion matrix.

	Predicted	Positive	Negative
Actual			
Positive		*TP*	*FN*
Negative		*FP*	*TN*

*T* means true, *F* means false, *P* means positive, *N* means negative.

**Table 5:** The weight of 11 types of features.

	Subjective Weights and Orders		Objective Weights and Orders		Comprehensive Weights and Orders		
(1)	(2)	(3)	(4)	(5)	(6)	(7)	(8)
Feature	Weights	Orders	Weights	Orders	Reasonable Value Range	Weights	Orders
X1	0.166	1	0.072	7	[0.072–0.166]	0.121	2
X2	0.142	3	0.109	4	[0.109–0.142]	0.125	1
X3	0.094	6	0.062	10	[0.062–0.094]	0.080	8
X4	0.031	10	0.071	8	[0.031–0.071]	0.053	11
X5	0.151	2	0.043	11	[0.043–0.151]	0.095	5
X6	0.140	4	0.102	5	[0.102–0.140]	0.120	3
X7	0.106	5	0.075	6	[0.075–0.106]	0.088	6
X8	0.041	8	0.069	9	[0.041–0.069]	0.056	10
X9	0.032	9	0.133	1	[0.032–0.133]	0.083	7
X10	0.023	11	0.132	3	[0.023–0.132]	0.073	9
X11	0.074	7	0.133	2	[0.074–0.133]	0.105	4

**Table 6:** The results of ensemble learning module with different parameters.

Loss Function	The Number of Base Learners	RMSE
Linear	30	10.2316
	50	9.6649
	70	9.3749
	100	10.0136
Square	30	9.9312
	50	10.3445
	70	10.1863
	100	9.3807
Exponential	30	10.2625
	50	9.7782
	70	10.0313
	100	9.8675

**Table 7:** Performance analysis for score indicators evaluated by the statistical modeling module.

**Statistical Modelling**	**Score Indicator**	**RMSE**
	Concentration	11.167
	Participation	13.409

**Table 8:** Performance analysis for category indicators evaluated by the statistical modeling module.

**Statistical Modelling**	**Category Indicators**		**Precision**	**Recall**	**F1**	**Accuracy**	**M_P**	**M_R**	**M_F1**
	Teachers’ Type	Indoctrination	0.987	0.938	0.962	0.815	0.789	0.791	0.790
		Natural	0.776	0.776	0.776				
		Interactive	0.604	0.659	0.630				
	Teachers’ Style	Passionate	0.982	0.918	0.949	0.695	0.703	0.687	0.695
		Humorous	0.511	0.414	0.457				
		Solemn	0.615	0.728	0.667				
	Teachers’ Media Usage	Multimedia	0.891			0.905	0.918	0.448	0.602
		Blackboard	0.919						

**Table 9:** Performance analysis for score indicators evaluated by the ensemble learning module.

**Ensemble Learning**	**Score Indicator**	**RMSE**
	Concentration	8.318
	Participation	9.375

**Table 10:** Performance analysis for category indicators evaluated by the ensemble learning model.

**Ensemble Learning**	**Category Indicators**		**Precision**	**Recall**	**F1**	**Accuracy**	**M_P**	**M_R**	**M_F1**
	Teachers’ Type	Indoctrination	0.947	0.935	0.941	0.785	0.755	0.761	0.758
		Natural	0.776	0.728	0.752				
		Interactive	0.542	0.619	0.619				
	Teachers’ Style	Passionate	0.951	0.935	0.943	0.73	0.729	0.719	0.724
		Humorous	0.578	0.464	0.515				
		Solemn	0.66	0.756	0.705				
	Teachers’ Media Usage	Multimedia	0.881			0.89	0.897	0.433	0.584
		Blackboard	0.899						

**Table 11:** The evaluation performance of all experiments.

**Statistical Modelling**		**RMSE**				**Overall Module Parameters**			
	Concentration	11.167							
	Participation	13.409	**Precision**	**Recall**	**F1**	**Accuracy**	**M_P**	**M_R**	**M_F1**
	Teachers’ Type	Indoctrination	0.987	0.938	0.962	0.815	0.789	0.791	0.790
		Natural	0.776	0.776	0.776				
		Interactive	0.604	0.659	0.630				
	Teachers’ Style	Passionate	0.982	0.918	0.949	0.695	0.703	0.687	0.695
		Humorous	0.511	0.414	0.457				
		Solemn	0.615	0.728	0.667				
	Teachers’ Media Usage	Multimedia	0.891			0.905	0.918	0.448	0.602
		Blackboard	0.919						
**Ensemble Learning**		**RMSE**				**Overall Module Parameters**			
	Concentration	8.318							
	Participation	9.375	**Precision**	**Recall**	**F1**	**Accuracy**	**M_P**	**M_R**	**M_F1**
	Teachers’ Type	Indoctrination	0.947	0.935	0.941	0.785	0.755	0.761	0.758
		Natural	0.776	0.728	0.752				
		Interactive	0.542	0.619	0.619				
	Teachers’ Style	Passionate	0.951	0.935	0.943	0.73	0.729	0.719	0.724
		Humorous	0.578	0.464	0.515				
		Solemn	0.66	0.756	0.705				
	Teachers’ Media Usage	Multimedia	0.881			0.89	0.897	0.433	0.584
		Blackboard	0.899						

## Data Availability

The data presented in this study are available on request from the corresponding author.

## Appendix A

### Appendix A.1.

The steps of calculating objective weights by the entropy weight method is as follow:

**Algorithms A1** Calculating objective weights by the entropy weight method	
**Input:** For totally *N* samples and *M* corresponding features, the *j*-th feature value of the *i*-th sample $x_{ij}\left(i=1,2,\ldots ,N;j=1,2,\ldots M\right)$;	
**Process:**	
1. $X_{ij}=\frac{x_{ij}-\min\left\{x_{1j},\cdots ,x_{nj}\right\}}{\max\left\{x_{1j},\cdots x_{nj}\right\}-\min\left\{x_{1j},\cdots ,x_{nj}\right\}}\;$	% Normalization of positive influence feature
2. $X_{ij}=\frac{\max\left\{x_{1j},\cdots ,x_{nj}\right\}-x_{ij}}{\max\left\{x_{1j},\cdots x_{nj}\right\}-\min\left\{x_{1j},\cdots ,x_{nj}\right\}}\;$	% Normalization of negative influence feature
3. $k=\frac{1}{\ln\left(n\right)}>0$, $e_{j}=-k\sum_{i=1}^{n}p_{ij}\ln\left(p_{ij}\right),j=1,2,\cdots ,m$	
	% Entropy value of the *j*-th feature
4. $d_{j}=1-e_{j},j=1,2,\cdots ,m$	% Information entropy redundancy of the *j*-th feature
**end** **Output:** Subjective weight of the *j*-th feature	

### Appendix A.2.

The specific learning process of the AdaBoost algorithm is as follow:

**Algorithms A2** Learning process of the AdaBoost algorithm	
**Input:****Dataset**$D_{1}\left(i\right)=1/m$**;**$D=\{(x_{1},y_{1}),(x_{2},y_{2}),\ldots ,(x_{m},y_{m})\}$; **Basic-learner**L; **Iteration**T;	
**Process:**	
1. $D_{1}\left(i\right)=1/m$;	% Initialize training set weight
2. for t=1,⋯,T:	
3. $h_{t}=L\left(D,D_{t}\right);$	% use *D* and *D_t_* to train the learner *h_t_*
4. $e_{t}={\mathrm{Pr}}_{x\sim D_{t,y}}\;I\left[h_{t}\left(x\right)\neq y\right]$;	% Calculate the error of learner *h_t_*
5. if $e_{t}>0.5$ then break	
6. $\alpha_{t}=\frac{1}{2}\ln\left(\frac{1-e_{t}}{e_{t}}\right)$;	% Calculate the coefficient of learner *h_t_*
7. $D_{t+1}\left(i\right)=\frac{D_{t}\left(i\right)}{Z_{t}}\times \{\begin{array}{c} \exp\left(-\alpha_{t}\right),h_{t}\left(x_{i}\right)=y_{i}\\ \exp\left(\alpha_{t}\right),h_{t}\left(x_{i}\right)\neq y_{i} \end{array}=\frac{D_{t}\left(i\right)\exp\left(-\alpha_{t}y_{i}h_{t}\left(x_{i}\right)\right)}{Z_{t}}$	
8. % Update the weight of training set, where *Z_t_* is the normalization factor.	
% $Z_{t}=\sum_{i=1}^{m}D_{i}\cdot \exp\left(-\alpha \cdot y_{i}\cdot L\left(x_{i}\right)\right)$	
9. end	
**Output:**$H\left(x\right)=sign\left(\sum_{t=1}^{T}\alpha_{t}h_{t}\left(x\right)\right)$	

## Appendix B

Original data come from real smart classrooms deployed by Hangzhou Hikvision Digital Technology Co. In a smart classroom, there is a pickup for voice acquisition, and two cameras recording videos for teachers and students, respectively.

List of data collection equipment in the real classroom is as follow.

**Device**	**Picture**	**Description**
Real Classroom		Overall layout of the smart classroom
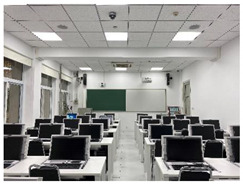
Pickup DS-2FP2020-A		To obtain the voice data in the classroom
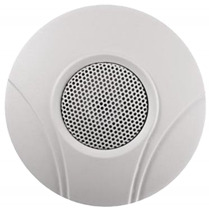
Camera for students iDS-ECD8012-H/T (8–32 mm)		In the front of the classroom. To record the voice for students, and obtain the data such as students’ movement, emotion…
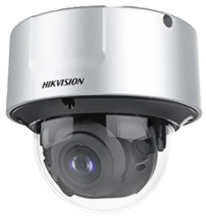
Camera for teachers iDS-EGD0288-HFR (8–32 mm) (2.8 mm)		In the middle of the classroom. To record the voice for teachers, and obtain the data such as teachers’ movement, emotion…
